# Digitized Morphometric Analysis using Maxillary Canine and Mandibular First Molar for Age Estimation in South Indian Population

**DOI:** 10.2174/1745017901814010762

**Published:** 2018-09-28

**Authors:** Vadivel Ilayaraja, Nalliapan Ganapathy, Georgebabu Jisha, Thambu Keerthipriyadharshini, Thangadurai Maheswaran, Thukanayakanpalayam R. Yoithapprabhunath

**Affiliations:** Department of Oral and Maxillofacial Pathology, Vivekanandha Dental College for Women, Tamilnadu, India

**Keywords:** Secondary dentin, Pulp tooth area ratio, Pulp chamber height, Age estimation, Digitalized orthopantomography, Maxillary Canine

## Abstract

**Background:**

In recent years, the estimation of age in living individuals have become important to solve judicial or civil problems. Secondary dentin deposition occurs as the age advances and can be measured by calculating the reduction in pulp chamber through Noninvasive radiograph techniques.

**Aim:**

The aim is to derive precise population specific formulae for age estimation.

**Materials and Methods:**

Digitalized Orthopantomography of 150 subjects, was retrieved. The subjects were divided into study and test group. Pulp tooth area ratio was recorded from the maxillary canine and pulp chamber crown root trunk height ratio was recorded from a mandibular first molar.

**Statistical Analysis:**

Regression equation was derived from study group subjects and this equation was used to estimate the age of subjects in the test group.

**Results:**

No significant differences were seen between mean chronological age and mean estimated age (*p*-value- 0.157). Mean Absolute Error (MAE) was estimated to be 2.76 years. The percentage of estimated ages in test sample < ± MAE was 72%. The difference was higher in the group I and II (*p*-value- 0.001 and 0.002)

**Conclusion:**

The equation derived from the anterior and posterior teeth together gave more accurate results in the present study. The prediction accuracy can further be enhanced by using multiple teeth or by utilizing other linear measurements in the same teeth.

## 1 INTRODUCTION

Age determination has become increasingly important in the field of forensics not only for the identification of dead but also for living individuals, to clarify legal queries in a matter of employment, labour acts, criminal offences, adoption, identification of immigrants, refugees and to determine the correct pensionable age and other similar purpose [1-3].

Over the years, teeth are being used for age assessment morphologically, histologically and radiographically, as it is the most durable and least affected by post-mortem changes [4]. Changes that are appreciable in teeth with increasing age are attrition, periodontal disease, secondary dentin deposition, root translucency, cementum apposition, root resorption, color changes, and increase in root roughness [2]. Identification of these changes requires tooth extraction, which is invasive and hence, it is impossible in living individuals. Considering these secondary changes with advancing age, various studies were done for age estimation of an individual. Secondary dentin formation being one of them develops within the pulp cavity; partly as a direct sign of aging and partly as a reaction against pathologic conditions like caries. Secondary dentin is deposited along the walls of pulp chamber leading to a decrease in the volume and size of the pulp chamber, which can be assessed and used in the estimation of age [2, 5]. Radiography being a non-invasive method plays a vital role in forensic dentistry to uncover the hidden facts which cannot be seen by means of physical examination [3, 6]. Inspection of radiographs and subsequent comparison with radiographic images, drawings, and descriptions in charts yields maturity scores that help us to assess the age of an individual [2, 7]. Therefore, radiographically estimating the reduction in pulp chamber size has been used over the years to determine the age of a living individual through various methods. Cameriere **et al**, derived age predicting regression equations using pulp tooth area ratio in mandibular canines, in an Italian sample (mean error 3-4.5 years). When Cameriere **et al**, used technique to derive an Indian specific equation, the mean absolute error of the predicted age was found to be more than 10 years [1, 8]. Errors lesser than ±10 years are only considered to be acceptable in forensic age prediction [2, 3, 8]. Saxena **et al**, obtained better accuracy in age prediction with Indian subjects using modified Kvaal’s method based on linear measurement of tooth, a technique based on linear measurements of pulp [6]. But all these methods involved anterior teeth, Matthew **et al**, developed an independent method in mandibular first molar for age estimation using pulp chamber crown trunk height ratio and obtained a higher accuracy with a mean error of 6.96 years [1]. Zou **et al**, stated that age can be estimated through regression analysis, which is an extension of simple linear regression used to assess the association between two or more independent variables and a single continuous dependent variable [9]. In the present study, both Cameriere **et al**, technique in maxillary canine and Matthew **et al**, method in mandibular first molar were used together to derive a regression equation, to determine the age of the individual.

## 2 AIM AND OBJECTIVES

The study was aimed to form a regression equation to estimate the age of individuals with minimum error margins for forensic age prediction.

## 3 MATERIALS AND METHODS

Digital Orthopantomograph (OPG) of 150 subjects was retrieved from the Department of Oral Medicine and Radiology, Vivekanandha dental college for women, Tiruchengode, Tamil Nadu. The subjects were divided into two groups, the study group (n=125) and the test group (n=25). The age of subjects in both the groups ranged from 17-60 years was grouped into Group I (17-20), Group II (20-29), Group III (30-39), Group IV (40-49) and Group V (50-60). The study group was used to find regression formulae to calculate the age from pulp tooth area ratio in the maxillary canine and pulp chamber crown trunk height ratio from a mandibular first molar. The test group was used to assess the accuracy of this formula.

### 3.1 Inclusion Criteria

1. OPG of subjects aged between 17 and 60 years.
2. The selected tooth on the OPG maxillary canine and mandibular first molar fully erupted into the oral cavity.
3. The root of the selected canine and molar was fully formed.

### 3.2 Exclusion Criteria

1. Teeth with any pathology, such as, Attrition, Caries or Periodontitis or periapical lesions, which would alter the surface area of the tooth.
2. Malaligned/rotated/ Impacted maxillary canines and mandibular first molar.
3. Tooth with filling or any prosthetic fittings.

### 3.3 Radiographic Measurements

**Fig. (1):**
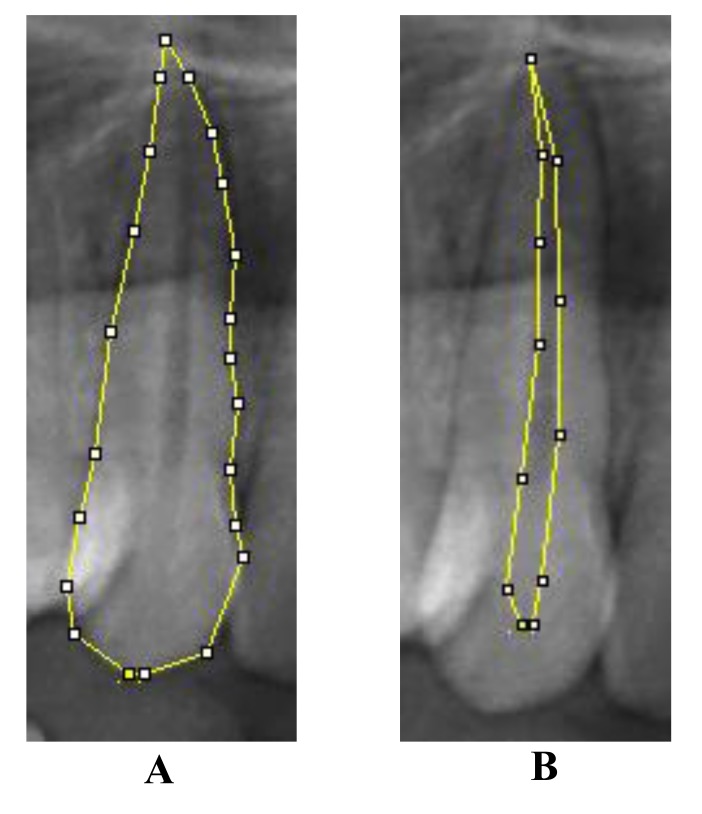
Tooth area and pulp area evaluation in maxillary canine by using Cameriere *et al*., method. **A).** 20 points were marked on tooth outline and joined to get the tooth area in maxillary canine. **B).** 10 points were marked on pulpal outline and joined to measure the pulpal area in maxillary canine.

Image J 1.46r (Wayne Rasband, National Institute of Health, USA) computer-aided drafting program was used to mark the points and record the measurements in the digitalized OPGs by two observers. Pulp tooth area ratio was measured in maxillary canine using Cameriere **et al*’s* method, in which 20 points were marked on the tooth outline and joined to get the tooth area. To measure the pulpal area, 10 points were marked on the pulpal outline and joined. Although few points were added in some cases for accurate measurements. Pulp tooth area ratio was calculated using these two measurements [10] (Fig. **1**).

**Fig. (2):**
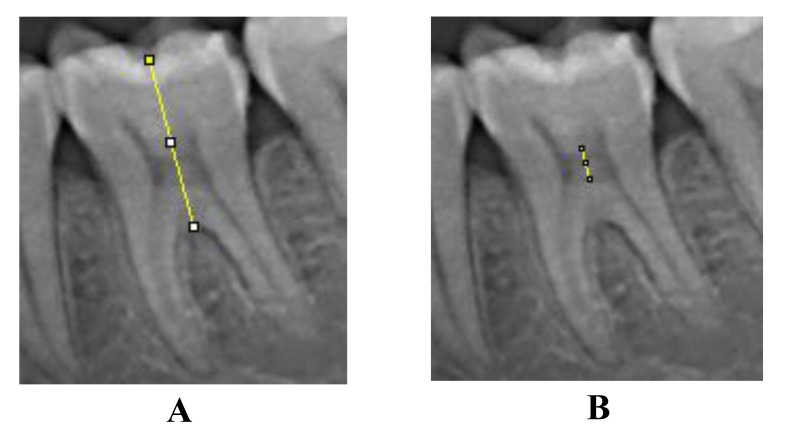
Crown trunk height and pulp chamber height evaluation in mandibular first molar using Matthew *et al* method. **A).** The distance between the central fossa and the highest point on the root furcation was recorded as crown root trunk height (CRTH). **B).** The distance between points on the roof and floor of pulp chamber was recorded as pulp chamber height (PCH).

Pulp Chamber Crown Trunk Height Ratio (PCTHR) was derived between pulp chamber height and crown root trunk height as described by Matthew **et al**., in a mandibular first molar. Points were marked on the central fossa and the highest point on the root furcation and a line was drawn connecting these points. The points on the roof and floor of pulp chamber were also marked and connected. The distance between the central fossa and the highest point on the root furcation was recorded as Crown Root Trunk Height (CRTH) and the distance between points on the roof and floor of pulp chamber was recorded as Pulp Chamber Height (PCH) [1] (Fig. **2**).

### 3.4 Statistical Analysis

All data were entered and analysed using a statistical software package (*SPSS*, version 16 for Windows). Pearson’s correlation coefficients were estimated to investigate the association between age and the calculated ratios from both the maxillary canine and mandibular first molar, respectively. A multiple regression equation was derived from the study group using Pulp tooth area ratio in the maxillary canine and Pulp chamber crown trunk height ratio in a mandibular first molar. This regression equation was applied to the test group to assess its accuracy in age estimation. The difference between the chronological age and estimated age was considered an error. The results were analysed using the unpaired *t*-test. Mean Absolute Error (MAE) was derived by calculating the mean of the absolute value of errors. Percentage of cases with the calculated age <± MAE and those falling within < ± 10 years, which is the error acceptable in forensic age prediction were calculated [8, 11]. A *p*-value <0.05 was considered to be statistically significant.

## 4 RESULTS

Orthopantomography (OPG) of 150 subjects was used for the study. In this study, the entire sample was distributed into five different age groups to observe the effect of this method on different age groups (Table **1**).

**Graph 1:**
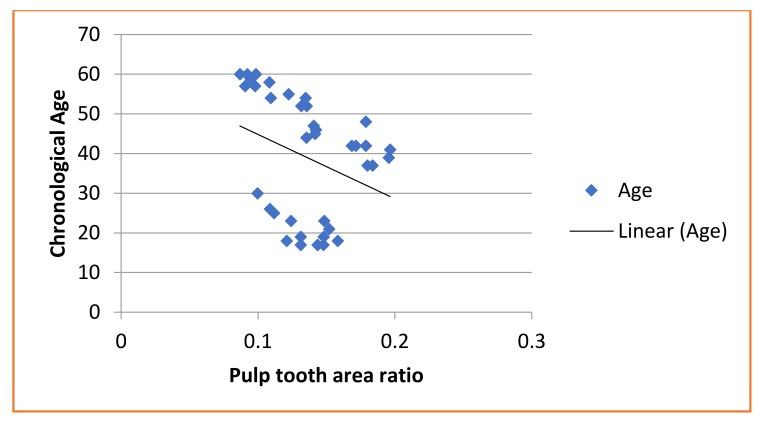
Scatter plot distribution of the pulp tooth ratio in maxillary canine when compared to the subject’s chronological age.

**Graph 2:**
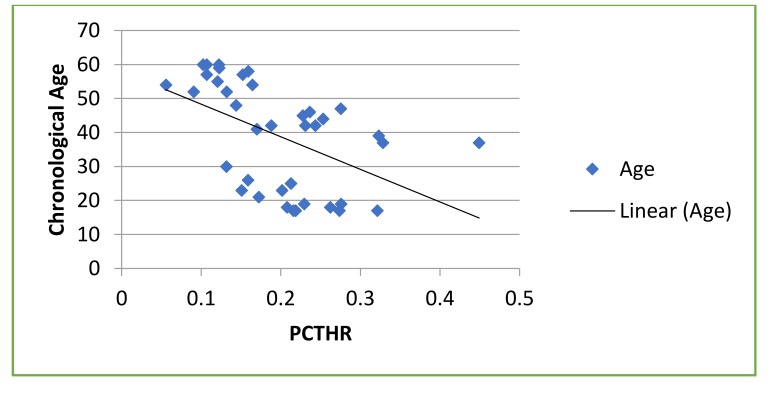
Scatter plot distribution of the pulp chamber height crown root trunk height ratio in mandibular first molar when compared to the subject’s chronological age.

A statistically significant negative correlation was found between chronological age and the ratios calculated in maxillary canine (R = –0.681; *P* < 0.001) and mandibular first molar (R = –0.594; *P* < 0.001), respectively (Table **2**, Graphs **1**-**2**).

Regression analysis on the study sample using pulp tooth area ratio in maxillary canine produced the following linear regression equation;

Age = −209.75(pulp tooth area ratio) + 63.877

Whereas, Pulp Chamber Crown Root Trunk Height Ratio (PCTHR) in mandibular first molar produced the following linear regression equation

Age = −98.053(PCTHR) + 55.682

The stepwise regression equation to estimate the age of individuals with minimum error margins for forensic age prediction is

Estimated age = 66.817−156.048(X1)−49.287(X2)

X1 = Pulp Tooth Area Ratio in maxillary canine

X2 = Pulp Chamber Crown Trunk Height Ratio in mandibular first molar

The R^2^ value for the regression equation is 0.522. To validate the accuracy of the estimation, the regression equations were applied to the test group and estimated age was calculated. Unpaired *t*-test was used to compare between the chronological age and estimated age in the test groups. No significant difference was found between mean chronological age and mean estimated age (*p*>0.05). MAE was estimated to be 2.76 years (Table **3**). The percentage of estimated ages in test sample falling within < ± 10 years was 72%. The difference between mean chronological age and mean estimated age in three age groups was within 10 years, the error acceptable in forensic age prediction. The difference was higher in the group I and II (*p*-value- 0.001 and 0.002), respectively.

## 5 DISCUSSION

Tooth is considered as a reliable body part for forensic age estimation since the environment has least influence on it. Age estimation through radiographs, by evaluating secondary dentin deposition is a non-invasive technique for adults [1-3]. In 1925, Bodecker established that the deposition of secondary dentin is correlated with age [12]. In 1995, Kvaal **et al**, presented a method for age estimation, which was based on an investigation of periapical radiographs [13], whereas Paewinsky **et al**, verified the applicability of this method on orthopantomograms [14]. In 2004, Cameriere **et al**, for the first time conducted a preliminary study to evaluate the variations in pulp/ tooth Area Ratio (AR) as an indicator of age and their method of age estimation seems promising [8, 10, 15, 16]. The method originally examined the maxillary canine but subsequently included the second molar and mandibular canine. While the authors obtained high levels of accuracy in age prediction (mean error -3 to 4.5 years), they advised that future research should investigate “the effect of race and culture in model parameters”. Indeed, others have also advocated the verification of age estimation methods on independent samples and some have concluded that best results are derived when population-specific formulas are used [17].

The present study, therefore, aimed at creating population specific formulae by using two teeth, maxillary canine and mandibular first molar. Maxillary canines were chosen as a representative for anterior teeth as Cameriere **et al**, stated that Canine are normally the oldest tooth, undergo less wear than other anterior tooth and are single-root with the largest pulp area and thus the easiest to analyze [16]. Mandibular first molars were chosen for posterior teeth as Hye-Mi Jeon **et al**., stated that OPGs show overlapping of premolars, superimposition structures in the incisor region. The maxillary posterior tooth is overshadowed by bone structure and Mandibular second molars frequently showed anatomical variation, particularly in Asians [18-20]. Different methods were used in both the teeth, for maxillary canines, Cameriere **et al**’s technique to evaluate pulp tooth area ratio used as the secondary dentin deposition occurs along all the walls of pulp chamber reducing the overall size, [21] whereas in mandibular molars, secondary deposition of dentin occurs preferentially on roof and floor, rather on the walls of the pulp chamber reducing the height instead of width, therefore Matthew **et al.**, method for measuring pulp chamber crown trunk height ratio was used [1]. There was no significant difference between the mean of estimated age and

mean of chronological age (*p*=0.157). In the present study, a significant difference was obtained in the subjects of group I and group II, (*p*-value- 0.001 and 0.002, respectively) shows that with advancing age, the size of the dental pulp cavity is reduced as a result of secondary dentin deposition [2]. In this study, there was a marked improvement in the MAE (2.76 years) when compared to earlier studies by, Jeevan **et al**, in which MAE of 4.28 years using pulp tooth area ratio in maxillary canine and by Matthew **et al**, using PCTHR alone in which MAE was 6.96 years [1, 22]. This shows that the regression equation derived by using both methods together has a greater accuracy with minimal error.

## CONCLUSION

The procedure using both anterior and posterior teeth together to derive a single regression equation gave more accurate results when compared to other methods used in the past. We can improve the prediction accuracy by deriving a multiple regression equation using multiple teeth or by utilizing other linear measurements in the same teeth reflecting secondary dentin deposition.

## Figures and Tables

**Table 1:** Age wise distribution of the subjects.

**Group**	**17-20 Years**	**21-29 Years**	**30-39 Years**	**40-49 Years**	**50-60 Years**	**Total**
Study group	25	25	25	25	25	125
Test group	5	5	5	5	5	25
Total	30	30	30	30	30	150

**Table 2:** Correlation of age with pulp tooth area ratio and molar chamber trunk ratio.

**Pearson Correlation with Age**	**Pulp Tooth Area Ratio in Maxillary Canine**	**Pulp Chamber Crown Trunk Height Ratio in Mandibular First Molar**
R	-0.681	-0.594

**Table 3:** Mean difference between actual age and estimated age in test group.

Groups	–	Mean	Standard Deviation	Significance
**Overall Test Group [N=25]**	Actual Age	34.8	12.6	0.157
	Estimated Age	37.56	5.862	
	MAE	2.764	1.892	
Test group 17-20 years [n=5]	Actual Age	19	1.225	0.001
	Estimated Age	31.21	3.404	
	MAE	12.212	3.415	
Test group 21-29 years [n=5]	Actual Age	24.6	2.608	0.002
	Estimated Age	36.28	4.442	
	MAE	11.682	3.566	
Test group 30-39 years [n=5]	Actual Age	33.6	3.05	0.375
	Estimated Age	36.17	5.928	
	MAE	2.566	5.751	
Test group 40-49 years [n=5]	Actual Age	45.6	2.074	0.167
	Estimated Age	42.01	4.005	
	MAE	3.59	4.765	
Test group 50-60 years [n=5]	Actual Age	51.2	2.168	0.011
	Estimated Age	42.15	4.213	
	MAE	9.05	4.55	
